# Animal Model of Acid-Reflux Esophagitis: Pathogenic Roles of Acid/Pepsin, Prostaglandins, and Amino Acids

**DOI:** 10.1155/2014/532594

**Published:** 2014-02-02

**Authors:** Koji Takeuchi, Kenji Nagahama

**Affiliations:** ^1^Division of Pathological Sciences, Department of Pharmacology and Experimental Therapeutics, Kyoto Pharmaceutical University, Misasagi, Yamashina, Kyoto 607-8414, Japan; ^2^General Incorporated Association, Kyoto Research Center for Gastrointestinal Diseases, Karasuma-Oike 671, Kyoto 604-8106, Japan

## Abstract

Esophagitis was induced in rats within 3 h by ligating both the pylorus and transitional region between the forestomach and glandular portion under ether anesthesia. This esophageal injury was prevented by the administration of acid suppressants and antipepsin drug and aggravated by exogenous pepsin. Damage was also aggravated by pretreatment with indomethacin and the selective COX-1 but not COX-2 inhibitor, whereas PGE_2_ showed a biphasic effect depending on the dose; a protection at low doses, and an aggravation at high doses, with both being mediated by EP1 receptors. Various amino acids also affected this esophagitis in different ways; L-alanine and L-glutamine had a deleterious effect, while L-arginine and glycine were highly protective, both due to yet unidentified mechanisms. It is assumed that acid/pepsin plays a major pathogenic role in this model of esophagitis; PGs derived from COX-1 are involved in mucosal defense of the esophagus; and some amino acids are protective against esophagitis. These findings also suggest a novel therapeutic approach in the treatment of esophagitis, in addition to acid suppressant therapy. The model introduced may be useful to test the protective effects of drugs on esophagitis and investigate the mucosal defense mechanism in the esophagus.

## 1. Introduction

Reflux esophagitis, an endoscopically positive gastroesophageal reflux disease, is mainly caused by excessive exposure to gastric contents due to impairments of various protective mechanisms that prevent reflux into the esophagus and resist the refluxate [1, 2]. Since gastric acid plays a key role in the pathogenesis of reflux esophagitis, luminal pH control is considered important in the management of this disease [2]. Antisecretory drugs, such as histamine H_2_ receptor antagonists and proton pump inhibitors, have been shown to be effective against acid-reflux esophagitis in humans and animals [3–5].

Pepsin, an acid-activated protease secreted by gastric chief cells, is also an important component of gastric refluxate into the esophagus, in addition to acid. Although there is currently no evidence for a definite role for pepsin in the pathogenesis of esophagitis [6], experimental evidence has demonstrated a pathogenic role for pepsin in the development of acute esophagitis models in rabbits or cats [7, 8]. However, the role of pepsin as an aggressive factor in the refluxate has not been studied in detail.

Nonsteroidal anti-inflammatory drugs (NSAIDs) are known to cause damage in the gastrointestinal mucosa and worsen the ulcerogenic response in these tissues [9, 10]. Adverse reactions to NSAIDs are mainly due to the inhibition of cyclooxygenase (COX) 1, the constitutive enzyme responsible for the production of prostaglandins (PGs) under normal conditions [11], although this paradigm has been challenged by the finding that PGs derived from COX-2 also play a role in maintaining the mucosal integrity of the gastrointestinal tract [12, 13]. However, the influences of NSAIDs and PGE_2_ on esophagitis have not yet been fully elucidated.

In this review, we introduced a rat model of acid-reflux esophagitis and described various pathogenic factors including aggressive factors such as acid and pepsin, as well as defensive factors such as prostaglandins (PGs) and nitric oxide (NO), mostly based on our previously published studies [14–17]. In addition, we showed the unique influences of various amino acids on this esophageal injury.

## 2. Induction of Acid-Reflux Esophagitis

**Figure 1:**
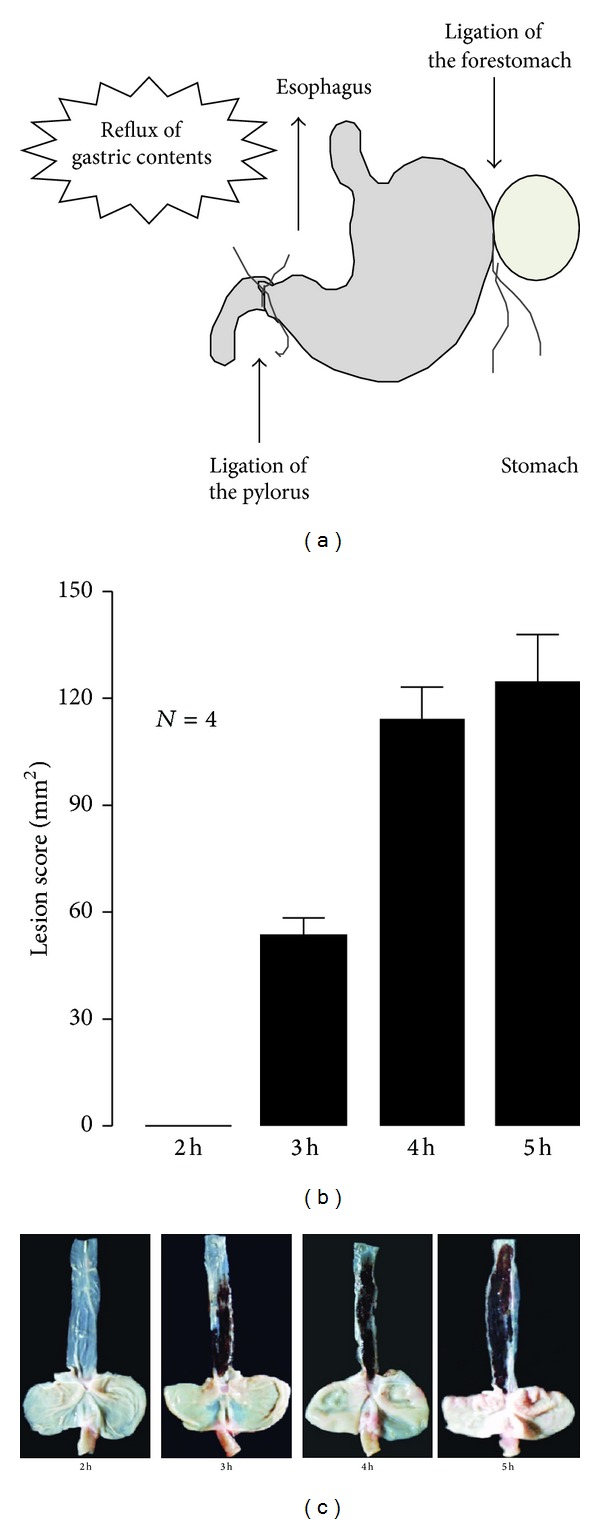
(a) Induction of acid-reflux esophagitis in rats. Under ether anesthesia, the abdomen was incised, and both the pylorus and junction between the corpus and forestomach were ligated. Three or four hours later, animals were killed by an overdose of ether, and the esophagus was removed, opened, and examined for hemorrhagic lesions. (b) Time-course changes in the development of acid-reflux esophagitis in rats. Under ether anesthesia, both the pylorus and forestomach were ligated, and the esophageal mucosa was examined 2~5 h later. Data were presented as the mean ± SE for 4 rats. (c) Gross appearance of esophageal lesions observed at 2, 3, 4, and 5 h after the ligation (from [14, 15] after modifications).

Rats were kept in individual cages with raised mesh bottoms and deprived of food but were allowed free access to tap water for 18 h prior to the experiments. Under ether anesthesia, the abdomen was incised along the middle, and then both the pylorus and junction between the forestomach and corpus were ligated [5] (Figure 1(a)). Following ligation of the pylorus and forestomach, severe hemorrhagic damage developed in the proximal 3 cm of the esophagus in a time-dependent manner (Figures 1(b) and 1(c)). Animals were autopsied 4 h after the double ligation to examine the protective effect of drugs and were autopsied 3 h after the ligation to examine the deleterious effect of drugs.

## 3. Importance of Acid and Pepsin in the Pathogenesis of Esophagitis

**Figure 2:**
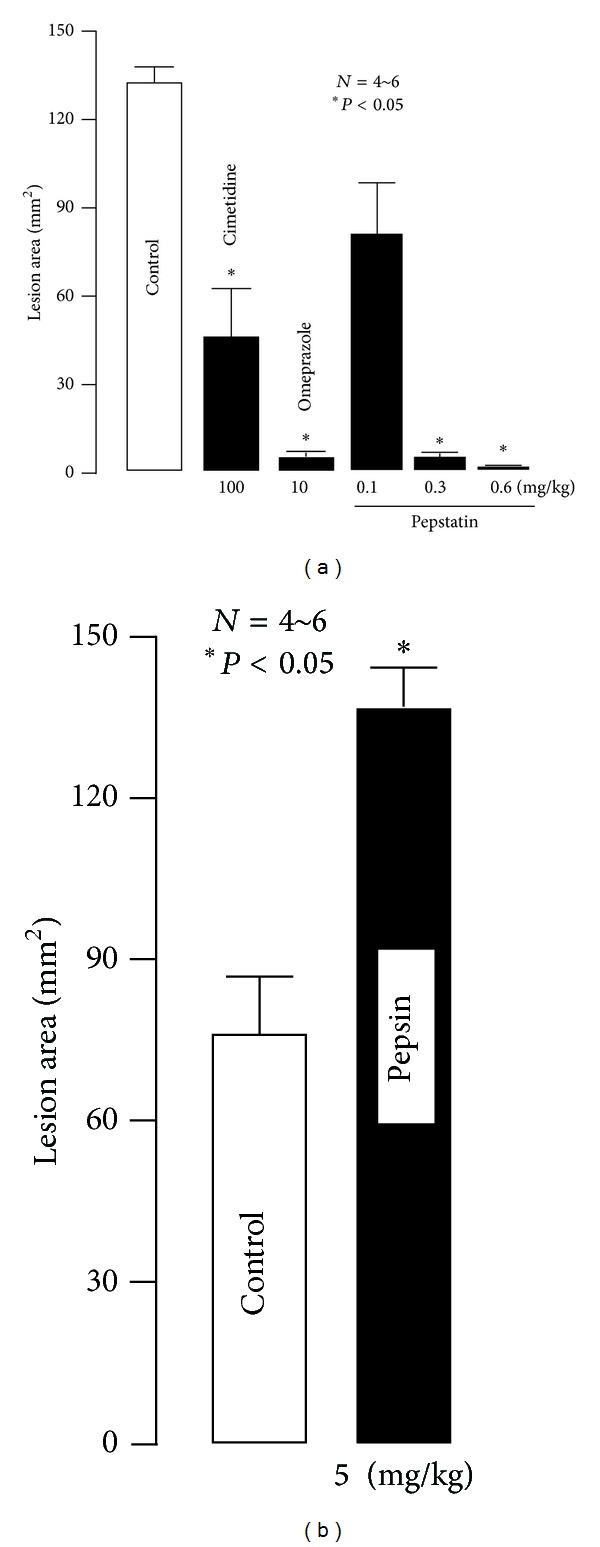
Effects of cimetidine, omeprazole, pepstatin (a), and pepsin (b) on acid-reflux esophagitis in rats. Under ether anesthesia, both the pylorus and forestomach were ligated, and the esophageal mucosa was examined 4 h later. Cimetidine (100 mg/kg) and omeprazole (10 mg/kg) were given i.d. immediately after the ligation, while pepstatin (0.1–0.6 mg/kg) and porcine pepsin (5 mg/kg) were given i.g. immediately after the ligation. Data were presented as the mean ± SE for 4~6 rats. *Significantly different from the control, at *P* < 0.05 (from [14, 15] after modifications).

The severity of acid-reflux esophagitis induced by double ligation of the pylorus and forestomach for 4 h was significantly reduced by the prior oral administration of omeprazole (10 mg/kg) or cimetidine (100 mg/kg) 30 min before the ligation (Figure 2(a)) [14, 15]. Likewise, pepstatin, a specific pepsin inhibitor (0.1~0.6 mg/kg), when administered intragastrically (i.g.), after the ligation, prevented the occurrence of these esophageal lesions in a dose-dependent manner, with inhibition at 0.3 mg/kg being almost 100% [15, 17]. The mucosal protective drug, ecabet Na (12.5 mg/kg, i.g.), also significantly prevented the development of these esophageal lesions. In contrast, porcine pepsin (5 mg/kg, i.g.) significantly aggravated the severity of esophageal damage induced by 3 h ligation of both the pylorus and forestomach (Figure 2(b)) [15]. Both omeprazole and cimetidine significantly decreased the output of acid and pepsin in pylorus-ligated rats, whereas pepstatin, even at 1 mg/kg, had no effect on acid output but completely inhibited pepsin output. Ecabet Na failed to affect pepsin output in the pylorus-ligated stomach; however, it inhibited the pepsin activity in the *in vitro* experiment [14, 15].

Reflux esophagitis is a chronic disease caused by the repeated contact of gastric contents with the esophageal epithelium. We observed that antisecretory drugs significantly prevented the development of esophageal lesions, which supported a key role of gastric acid in the pathogenesis of esophageal lesions [14, 15]. It is known that gastroesophageal reflux has been established as a major risk factor for esophageal adenocarcinoma and Barrett's esophagus and that acid exposure has pro-proliferative and antiapoptotic effects which may facilitate neoplastic progression of Barrett's esophagus [18]. In this sense, acid suppression serves not only as an effective prophylactic mean for the reflux esophagitis but also as a potential chemopreventive strategy for Barrett's esophagus. In addition to gastric acid, pepsin, conjugated or deconjugated bile acids, and pancreatic enzymes are also included in the refluxate into the esophagus. However, it is unlikely that bile acids and pancreatic enzymes participated in the pathogenesis observed in the present rat esophagitis model because this model was induced in pylorus-ligated stomachs in which no regurgitation occurred from the duodenal contents into the stomach. Interestingly, pepstatin potently prevented the occurrence of acid-reflux esophagitis [15]. This drug has been shown to exhibit potent inhibitory activity against the proteolytic activity of pepsin [19] and complete protection against the forestomach ulceration induced by pylorus ligation [20]. Since this agent did not cause any influence on gastric juice volume or acid output at the dose that prevented esophageal lesions in the present model, it is likely that the protective effect of pepstatin was due to the inhibition of pepsin activity. Similar protection was observed with ecabet Na, the agent known to exhibit the antipeptic action [15]. Ecabet Na is known to inhibit pepsin activity through interaction with the substrate *in vitro* [15, 21]. We also observed that exogenous administered pepsin significantly which worsened the severity of esophageal lesions in the present model. These results strongly suggest that pepsin plays a major role in the pathogenesis of acid-reflux esophagitis. Certainly, there is a possibility that gastricsin may play a pathogenic role in this esophagitis model, although the proteolytic action is very weak. Although esophagitis is caused by duodenogastric reflux consisting of retrograde passage of alkaline duodenal contents (bile and pancreatic juice) into the stomach [22, 23], it is unlikely that trypsin is involved in the pathogenesis of this model, because the pancreatic juice cannot regurgitate into the esophagus through the pylorus-ligated stomach.

## 4. Effect of Various COX Inhibitors and PGE_2_ on Esophagitis

**Figure 3:**
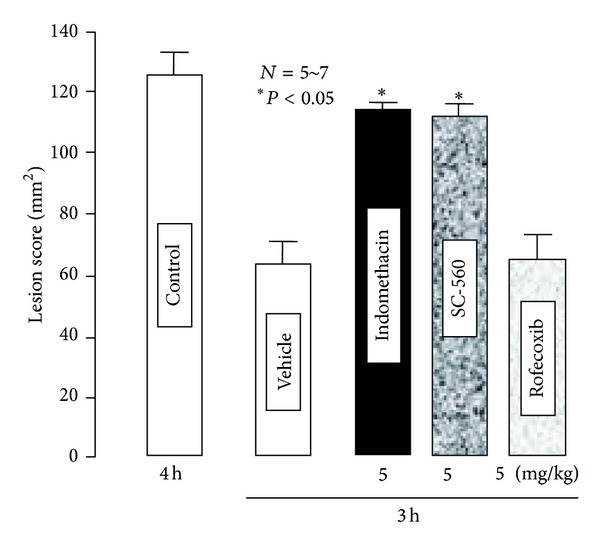
Effects of various NSAIDs on acid-reflux esophagitis in rats. Esophagitis was induced by ligation of both the pylorus and forestomach, and animals were killed 4 h later. Indomethacin (a nonselective CO inhibitor), SC-560 (a selective COX-1 inhibitor), or rofecoxib (a selective COX-2 inhibitor) was given i.d. at a dose of 5 mg/kg 30 min before the double ligation. Data are presented as the mean ± SE for 5~7 rats. *Significantly different from the control, at *P* < 0.05 (from [16] after modifications).

**Figure 4:**
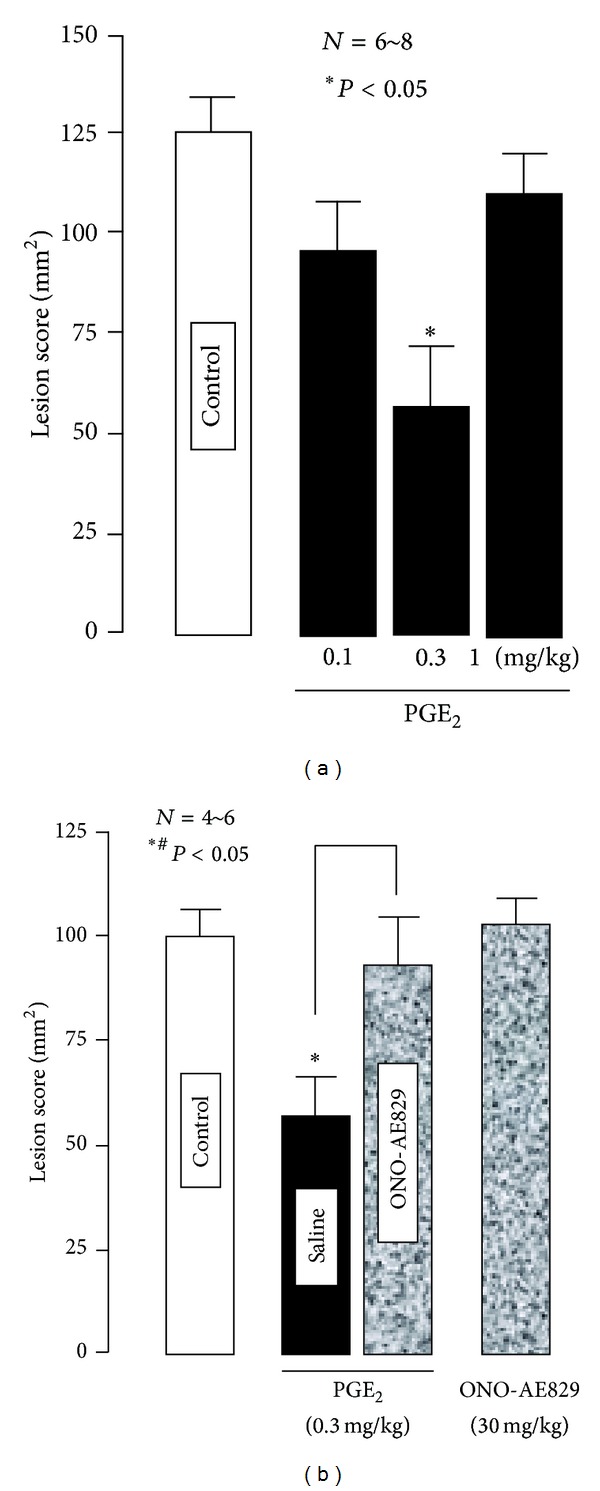
Effects of PGE_2_ on acid-reflux esophagitis (a) and its attenuation by ONO-AE-829, an EP1 antagonist (b), in rats. Esophagitis was induced by ligation of both the pylorus and forestomach, and animals were killed 4 h later. PGE_2_ (0.1~1 mg/kg) was given i.v. 10 min before the double ligation, while ONO-AE-829 (30 mg/kg) was given s.c. 30 min before the administration of PGE_2_. Data are presented as the mean ± SE for 4~8 rats. Significant difference at *P* < 0.05; *from the control; ^#^from PGE_2_ alone (from [16] after modifications).

**Figure 5:**
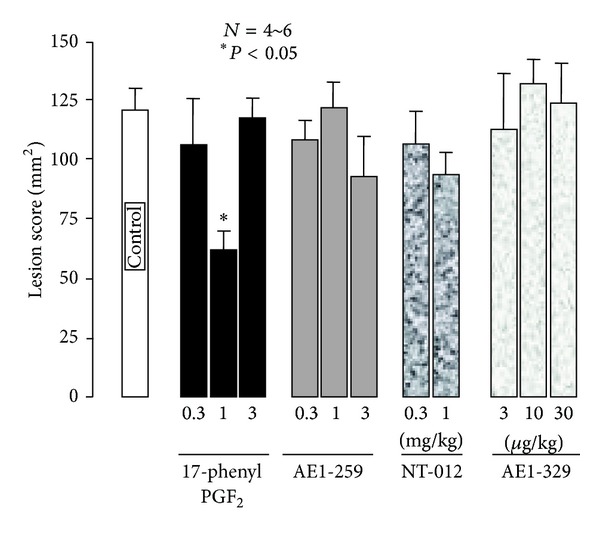
Effects of various selective EP receptor agonists on esophageal lesions in rats. Esophagitis was induced by ligation of both the pylorus and forestomach, and animals were killed 3 h later. 17-Phenyl PGE_2_ (EP1 agonist: 0.3~3 mg/kg), ONO-AE1-259 (EP2 agonist: 0.1~1 mg/kg), ONO-NT-012 (EP3 agonist: 0.3 and 1 mg/kg), and ONO-AE1-329 (EP4 agonist: 1~30 *μ*g/kg) were given i.v. 10 min before the double ligation. The esophageal mucosa was examined 4 h later. Data are presented as the mean ± SE for 4~6 rats. *Significantly different from the control, at *P* < 0.05 (from [16] after modifications).

The severity of these lesions was significantly aggravated when the animals were pretreated with indomethacin (5 mg/kg) given intraduodenally (i.d.) 30 min before double ligation of the pylorus and forestomach (Figure 3). This response was mimicked by the selective COX-1 inhibitor SC-560 (5 mg/kg, i.d.) but not by the selective COX-2 inhibitor rofecoxib (5 mg/kg, i.d.) [16]. On the other hand, PGE_2_ given intravenously (i.v.) 10 min before the double ligation had a biphasic effect on the esophageal lesions induced by ligation of both the pylorus and forestomach; this agent prevented the occurrence of esophageal lesions in a dose-dependent manner at lower doses (0.1 and 0.3 mg/kg), while the effect disappeared when the dose was increased to 1 mg/kg (Figure 4(a)). The protective effect of PGE_2_ at 0.3 mg/kg was significantly abrogated by the prior administration of ONO-AE-829 (30 mg/kg), the EP1 antagonist, given subcutaneously (s.c.) (Figure 4(b)). As expected, 17-phenyl PGE_2_ (EP1 agonist; 0.3–3 mg/kg, i.v.) also had a biphasic effect on the severity of esophageal damage, similar to PGE_2_ (Figure 5). Neither ONO-AE1-259 (EP2 agonist; 0.1–1 mg/kg), ONO-NT-012 (EP3 agonist; 0.3 and 1 mg/kg), nor ONO-AE1-329 (EP4 agonist; 3–30 *μ*g/kg) given i.v. had a significant effect on the development of esophageal lesions.

**Figure 6:**
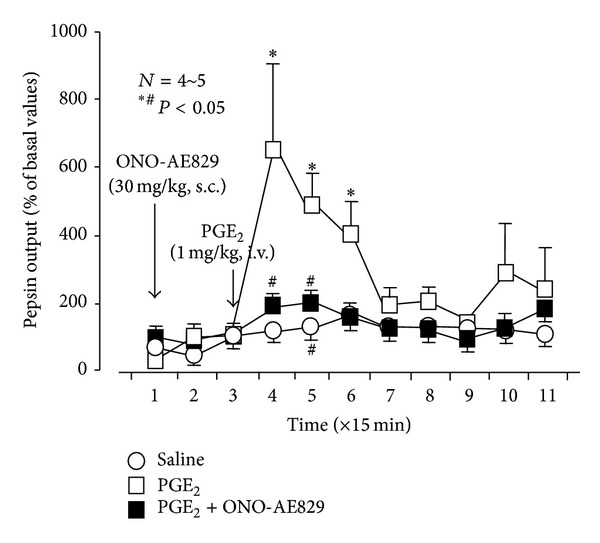
Influence of ONO-AE-829 on the stimulatory effect of PGE_2_ on pepsin secretion in rats under urethane anesthesia. An acute fistula stomach was filled with 2 mL of saline through the fistula, and the solution was changed every 15 min. PGE_2_ (1 mg/kg) was given i.v. 30 min after the start of the experiment, while ONO-AE-829 (30 mg/kg) was given s.c. 1 h before the administration of PGE_2_. Data are presented as the mean ± SE of values determined every 15 min for 4~5 rats. Significant difference at *P* < 0.05; *from saline; ^#^from PGE_2_ alone.

Neither indomethacin, SC-560, nor rofecoxib had any effect on the secretion of acid and pepsin in pylorus-ligated rats [16]. Although PGE_2_ did not significantly affect acid output in pylorus-ligated stomachs at any dose, it increased pepsin output in a dose-dependent manner. The same effect was reproduced by 17-phenyl PGE_2_ (EP1 agonist), whereas neither ONO-AE1-259 (EP2 agonist), ONO-NT-012 (EP3 agonist), nor ONO-AE1-329 (EP4 agonist) had any effect on gastric secretion, in terms of acid or pepsin output. The stimulatory effect of PGE_2_ on pepsin secretion was also confirmed in urethane-anesthetized rats. The secretion of pepsin was markedly increased after the administration of PGE_2_ (1 mg/kg, i.v.) and reached a maximal level 15 min later; however, this effect was not observed in animals pretreated with the EP1 antagonist ONO-AE-829 (30 mg/kg, s.c.) (Figure 6).

We demonstrated that acid-reflux esophagitis was markedly aggravated by indomethacin as well as SC-560, but not rofecoxib [16]. These findings suggest the importance of endogenous PGs in defense of the esophageal mucosa against acid injury and that such protective PGs are mainly derived from COX-1, not COX-2. Goyal [24] reported that indomethacin reduced the severity of esophageal lesions in rabbits, which indicated a deleterious effect of PGs on the esophageal mucosa. Northway et al. [25] reported that indomethacin prevented radiation-induced esophagitis in the opossum. The difference in results between their study and ours may be due to differences in the experimental conditions such as the animal species and esophagitis models used. Interestingly, PGE_2_ was found to have a biphasic influence on the development of acid-reflux esophageal lesions; a protective effect at lower doses; and a deleterious effect at a high dose, and both effects were mediated by the activation of EP1 receptors [16]. Concerning the protective action, the same effect was achieved with 17-phenyl PGE_2_ (EP1 agonist), but not with other prostanoids including the EP2 agonist, EP3 agonist, and EP4 agonist. These results strongly suggest the involvement of EP1 receptors in the protective action of PGE_2_ against esophageal lesions. This was further supported by the finding that ONO-AE-829, a selective EP1 antagonist, significantly attenuated the protective effect of PGE_2_ on esophageal damage.

We previously reported that PGE_2_ afforded gastric protection by modulating various functions mediated by the activation of EP1 receptors, such as stimulating HCO_3_^−^ secretion, inhibiting motility, or increasing mucosal blood flow [26–29]. In addition, endogenous PGs are known to mediate the gastric hyperemic response to an acid challenge in damaged stomachs and help maintain mucosal integrity under such adverse conditions [28]. This hyperemic response was previously shown to occur primarily through capsaicin-sensitive afferent neurons [30], yet endogenous PGs contribute to this response by sensitizing these afferent neurons through the activation of EP1 receptors [29]. The same may be true in the esophagus after the reflux of an excessive amount of gastric acid, and PGs may act as a mediator of such a protective function in this tissue, in collaboration of these afferent neurons. Further studies are needed to elucidate the mechanism by which PGE_2_ protects the esophageal mucosa against acid injury.

Interestingly, PGE_2_ exhibited a protective effect on acid-reflux esophageal damage through EP1 receptors yet may have a deleterious effect on these lesions via the same receptor. This biphasic effect of PGE_2_ was evident when the protective effect observed at 0.3 mg/kg disappeared once the dose was increased to 1 mg/kg. The same effect was also observed after the administration of 17-phenyl PGE_2_, the EP1 agonist. It is assumed that PGE_2_ exerts both protective and deleterious actions; however, the protective action overcomes the deleterious action at low doses, resulting in protection against esophageal injury, while these opposite actions are in balance at high doses, resulting in no protection or aggravation. The question of why PGE_2_ has a deleterious influence on esophageal damage then arises. We found that both PGE_2_ and 17-phenyl PGE_2_ dose-dependently increased the secretion of pepsin in pylorus-ligated stomachs, which suggested the involvement of EP1 receptors in PGE_2_-induced pepsin secretion [16]. This was supported by the finding that the stimulatory effect of PGE_2_ on pepsin was completely attenuated by ONO-AE-829, the EP1 antagonist. Thus, these prostanoids may irritate the esophageal mucosa by stimulating the secretion of pepsin via EP1 receptors. Most previous studies showed the inhibitory effect of PGE_2_ and its derivatives on pepsin secretion [7]; however, its stimulatory action was reported by Defize and Hunt [31], who showed that both PGE_1_ and PGE_2_ increased the secretion of pepsinogen *in vitro *using canine chief cell monolayer cultures. The present study confirmed their findings and further showed the involvement of EP1 receptors in the stimulatory action of PGE_2_ on pepsin secretion.

## 5. Effect of Various Amino Acids on Acid-Reflux Esophagitis

**Figure 7:**
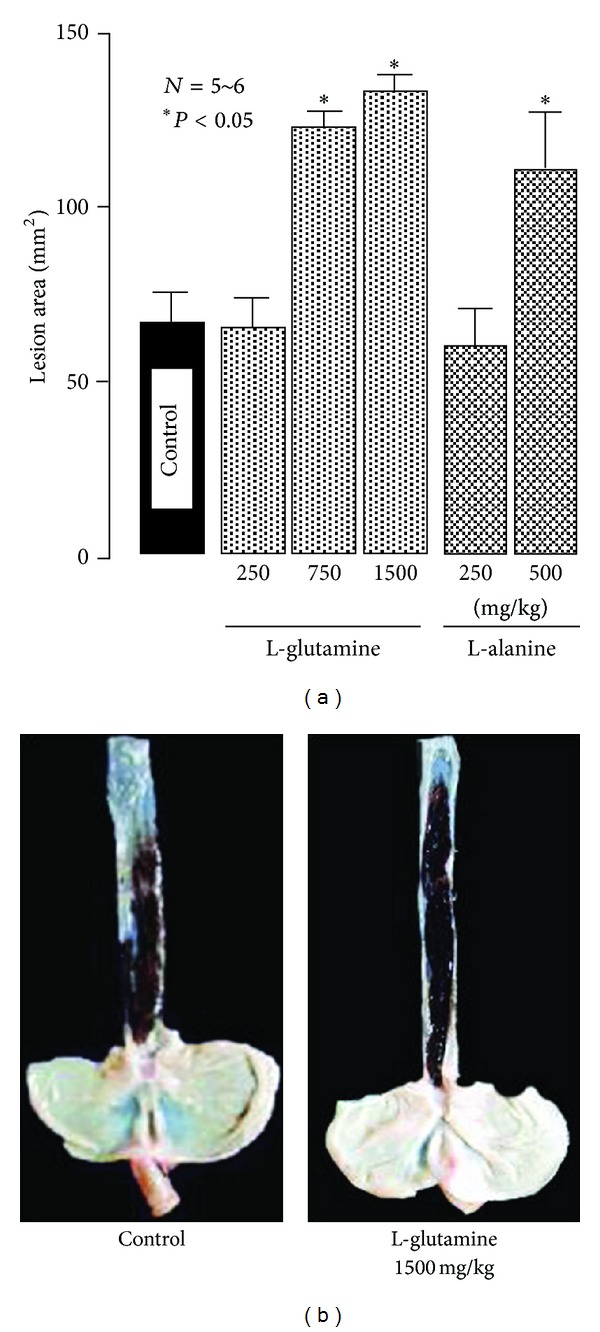
Effects of L-glutamine and L-alanine on acid-reflux esophagitis in rats. Under ether anesthesia, both the pylorus and forestomach were ligated, and the esophageal mucosa was examined 3 h later. L-glutamine (250–1500 mg/kg) and L-alanine (250 and 500 mg/kg) were given i.g. immediately after the ligation. Data are presented as the mean ± SE for 5~6 rats. *Significantly different from the corresponding control at *P* < 0.05. (b) Macroscopic appearance of esophageal lesions induced by ligation of both the pylorus and forestomach for 3 h. L-glutamine (1500 mg/kg) was given i.g. immediately after the ligation. Note that L-glutamine apparently aggravated the severity of hemorrhagic esophageal lesions (from [15, 17] after modifications).

**Figure 8:**
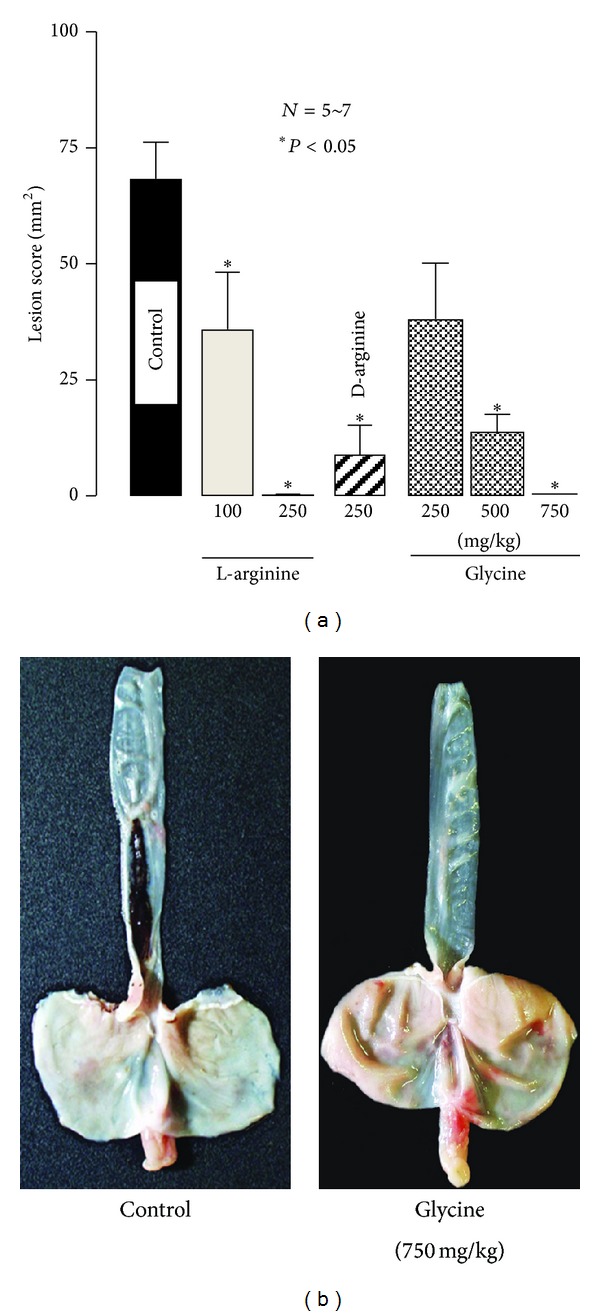
Effects of L- or D-arginine and glycine on acid-reflux esophagitis in rats. Under ether anesthesia, both the pylorus and forestomach were ligated, and the esophageal mucosa was examined 4 h later. L-arginine (100 and 250 mg/kg), D-arginine (250 mg/kg), or glycine (250–750 mg/kg) was given i.g. immediately after the ligation. Data are presented as the mean ± SE for 5~7 rats. *Significantly different from the control, at *P* < 0.05. (b) Macroscopic appearance of esophageal lesions induced by the dual ligation for 3 h. Glycine (750 mg/kg) was given i.g. immediately after the ligation. Note that glycine completely inhibited the development of hemorrhagic esophageal lesions (from [17] after modifications).

**Figure 9:**
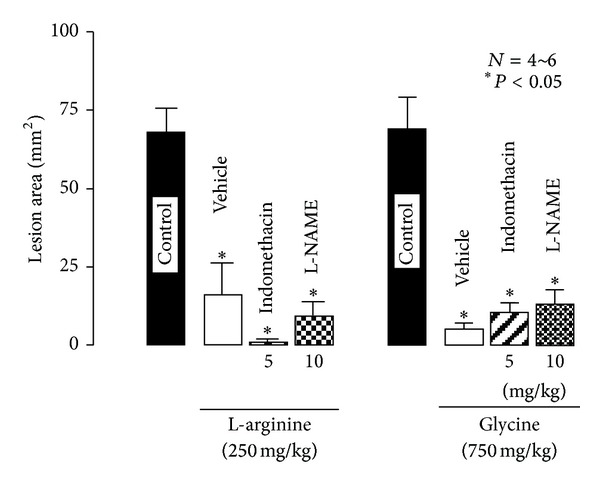
Effects of indomethacin and L-NAME on the protective action of L-arginine or glycine against acid-reflux esophagitis in rats. Under ether anesthesia, both the pylorus and forestomach were ligated, and the esophageal mucosa was examined 3 h later. L-arginine (250 mg/kg) or glycine (750 mg/kg) was given i.g. immediately after the ligation. Indomethacin (5 mg/kg) or L-NAME (10 mg/kg) was given s.c. 30 min before the ligation. Data are presented as the mean ± SE for 4~6 rats. *Significantly different from the control, at *P* < 0.05 (from [17] after modifications).

The intragastric administration of L-glutamine (250~1500 mg/kg) dose-dependently increased the severity of esophageal lesions, and a significant effect was observed at 750 mg/kg or greater; the degree of aggravation at 1500 mg/kg was over 200% (Figures 7(a) and 7(b)) [15]. Similarly, L-alanine significantly worsened the severity of lesions at 500 mg/kg, with the lesion score being almost 2 times the control value [17]. In contrast, L-arginine (100 and 250 mg/kg, i.g.) reduced the severity of esophageal lesions, with complete inhibition being observed at 250 mg/kg, and the same effect was obtained by D-arginine (250 mg/kg, i.g.) [17] (Figure 8(a)). Glycine (250~750 mg/kg, i.g.) also dose-dependently reduced the severity of esophagitis (Figures 8(a) and 8(b)). The protective action of L-arginine or glycine was not significantly affected by the pretreatment of animals with indomethacin (5 mg/kg, s.c.) or L-NAME (10 mg/kg, s.c.) (Figure 9).

Since the amino acids used had different effects, protective or aggravative, on acid-reflux esophagitis, and because the severity of esophagitis was influenced by the pH of the gastric contents, their effects on gastric pH may also be different after i.g. administration. To investigate this possibility, we examined the effect of these amino acids on the pH of the gastric contents in pylorus-ligated rats [17]. Ligation of the pylorus for 3 h accumulated about 6 mL of gastric juice in the stomach, with the pH of the contents being 1.30 ± 0.05. Although none of the amino acids (L-arginine, L-alanine, L-glutamine, or glycine) at the doses used significantly affected the volume of gastric contents, the pH of the gastric contents was significantly increased by these amino acids. Notably, L-alanine at 500 mg/kg and glycine at 750 mg/kg increased the pH to 2.36 ± 0.12 and 2.38 ± 0.07, respectively. Likewise, both L-arginine at 250 mg/kg and L-glutamine at 750 mg/kg significantly raised the pH of the gastric contents to 1.82 ± 0.05 and 1.73 ± 0.09, respectively.

**Figure 10:**
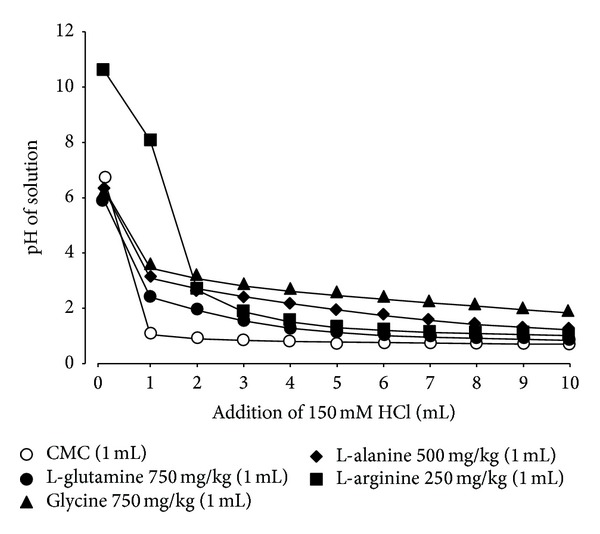
Buffering capability of various amino acids against HCl *in vitro*. L-alanine (500 mg/kg), L-arginine (250 mg/kg), L-glutamine (750 mg/kg), or glycine (750 mg/kg) was suspended or dissolved in a 0.5% CMC solution, and 1 mL of these solutions was titrated by the addition of 150 mM HCl. Changes in the pH of the solution were determined by a pH meter (from [17] after modifications).

Since the proteolytic activity of pepsin has been shown to be dependent on pH and maximal at approximately pH 2.0, and because pepsin plays an important role in the pathogenesis of acid reflux esophagitis [8, 14, 15], the different effects of amino acids on acid reflux esophagitis may be attributable to differences in their acid-buffering capability to modify the optimal pH for the proteolytic action of pepsin. To investigate this possibility, we titrated the solution of amino acids while varying the pH with the addition of HCl *in vitro*. The solution of L-arginine was a strong base, pH 10.6, whereas that of other amino acids was neutral, pH 6~7. When these solutions were titrated with the addition of 150 mM HCl, the amino acids exhibited a similar buffering action against HCl, although the potency was slightly different depending on the dose used (Figure 10). No significant difference was observed in the buffering capabilities of the amino acids used at around pH 2, the optimal pH for pepsin activity.

The intragastric administration of glycine or L-arginine was found to potently inhibit the acid-reflux esophagitis induced by the dual ligation, while L-alanine as well as L-glutamine aggravated the lesions. In contrast, L-glutamine worsened the severity of esophagitis by increasing the proteolytic activity of pepsin in the refluxate through a shift in the intraluminal pH to around 2.0, the optimal pH for peptic activity [15]. This amino acid has been shown to aggravate Shay ulceration in the forestomach caused by pylorus ligation in rats [32]. Since this model is caused by the corrosive actions of acid and pepsin, and because the esophageal mucosa is covered by stratified squamous epithelium, similar to the epithelium in the forestomach, it would be understandable for the mechanism aggravating these lesions to be associated with peptic activity. If aggravation is really induced by such a buffering capability, then other amino acids would be similarly expected to aggravate esophageal lesions. However, aggravation was observed with the intragastric administration of L-alanine, while other amino acids, such as L-arginine and glycine, markedly protected against esophageal lesions. We also found that these amino acids had a potent buffering action and increased luminal pH to around 2.0, similar to L-alanine or L-glutamine. Thus, it is assumed that L-arginine and glycine exert a protective effect against acid-reflux esophagitis due to yet unknown mechanisms, in spite of the increase in luminal pH and probably pepsin activity, similar to L-glutamine.

Acid-reflux esophagitis was previously shown to be aggravated by indomethacin and SC-560, but not rofecoxib, which suggested the participation of COX-1/PGE_2_ in the mucosal defense against esophagitis [16]. PGE_2_ prevented the development of acid-reflux esophagitis. In addition, L- or D-arginine given p.o. acted as a mild irritant and afforded a cytoprotective action against HCl-induced gastric lesions mediated by endogenous PGs [33]. It is possible that L-arginine and glycine may prevent the development of esophagitis via adaptive cytoprotection. However, the protective effect of these amino acids was not influenced by indomethacin, which excluded the possibility that endogenous PGs were involved in the protective action of these amino acids in the esophageal mucosa [17].

NO is known to regulate various biological processes in the gastrointestinal tract [34, 35]. Since L-arginine is a substrate for NO production, it is possible that the protective effect of this amino acid is partly mediated by NO. However, the role of NO in the pathogenesis of esophagitis remains controversial [7, 36–38]. Ozel et al. [36] reported that the rabbit esophageal mucosa exhibited mucosal adaptation to acid and pepsin, which was at least partly mediated by a NO-dependent mechanism. Ishiyama et al. [37] showed that exogenous NO exacerbated tissue damage in a reflux esophagitis model of rats. We recently demonstrated that NO increased pepsinogen secretion in the rat stomach via the stimulation of guanylyl cyclase [38]. In the present study, we found that the protective effect of L-arginine as well as glycine was not significantly antagonized by L-NAME, a NO synthase inhibitor. Thus, it is unlikely that these amino acids afford protection against acid-reflux esophagitis mediated by a NO-dependent mechanism. This is supported by the finding that D-arginine had a similar protective effect to L-arginine at the same dose because the former amino acid cannot be used as a substrate for NO production. Further studies are necessary to elucidate the mechanism underlying the protective action of L-arginine or glycine, in spite of that both of these amino acids exhibit a strong buffering capability, similar to L-glutamine.

Recent studies demonstrated that the esophagus has mechanisms to defend against damage from the refluxate, particularly gastric acid and pepsin, including an antireflux barrier (the lower esophageal sphincter), luminal clearance, and tissue resistance, increased cell replication, and increased blood supply to the esophagus [29, 39–41]. It is assumed that reflux esophagitis is due to impairments in epithelial defense against acid-pepsin contact [42, 43]. The mechanisms of these phenomena are not well defined; however, they may be mediated, at least partly, by endogenous PGs and NO as well as capsaicin-sensitive afferent neurons [7, 14, 16, 44]. Since both L-arginine and glycine exhibited protection against acid-reflux esophagitis in the presence of indomethacin or L-NAME, it is unlikely that such protective actions are mediated by endogenous PGs or NO. It is possible that these amino acids prevent acid-reflux esophagitis via the amelioration of defensive mechanisms that are mediated by factors other than PGs and NO.

## 6. Summary and Future Prospects

The results introduced in this review suggest that (1) acid/pepsin plays a major pathogenic role in the development of acid-reflux esophagitis, (2) endogenous PGs derived from COX-1 are involved in the mucosal defense of the esophagus, (3) exogenous PGE_2_ exerts a biphasic influence on acid-reflux esophagitis depending on the dose; a protective effect at low doses; and a deleterious effect at high doses, with both being mediated by EP1 receptors, and (4) various amino acids affect the severity of esophagitis in different ways, due to yet unidentified mechanisms; L-alanine and L-glutamine exert a deleterious effect on esophagitis, while L-arginine and glycine are highly protective and independent of endogenous PGs and NO. These findings may contribute to the development of a novel therapeutic approach for the treatment of reflux esophagitis, in addition to acid suppressant therapy. Souza et al. [45] suggested that esophagitis might not appear until weeks after the induction of reflux in rat models; in the development of reflux esophagitis the refluxed gastric juice does not directly damage the esophagus but rather stimulates esophageal epithelial cells to secrete chemokines that mediate damage of esophageal tissue. Although in the present esophagitis model the damage occurred 3 h after the onset of acid reflux, the acid-induced chemokine section might also be involved in the pathogenesis of acutely occurred acid-reflux esophagitis. Finally, the experimental esophagitis model introduced here is different from human reflux esophagitis, yet this model may be useful for testing protective effects of drugs on esophagitis and investigating the mucosal defense mechanism against esophageal injury. Certainly, because the difficult comparison in the pathophysiology of esophagitis exists between human and animals, there should be limitation for application of the present findings in human.
